# Accuracy of self-reported foot strike pattern detection among endurance runners

**DOI:** 10.3389/fspor.2024.1491486

**Published:** 2024-12-11

**Authors:** Heather K. Vincent, Kyle Coffey, Aiden Villasuso, Kevin R. Vincent, Sharareh Sharififar, Lydia Pezzullo, Ryan M. Nixon

**Affiliations:** ^1^ Exercise and Functional Fitness Laboratory, Department of Physical Medicine and Rehabilitation, University of Florida, Gainesville, FL, United States; ^2^ Department of Applied Physiology and Kinesiology, University of Florida, Gainesville, FL, United States

**Keywords:** running, foot strike, running shoe, biomechanics, gait

## Abstract

**Introduction:**

Foot strike pattern is often associated with running related injury and the focus of training and rehabilitation for athletes. The ability to modify foot strike pattern depends on awareness of foot strike pattern before being able to attempt change the pattern. Accurate foot strike pattern detection may help prevent running related injury (RRI) and facilitate gait modifications and shoe transitions. The purposes of this study were to determine the accuracy of self-reported foot strike pattern among endurance runners, to identify what factors were predictive of accurate foot strike detection and recent RRI.

**Methods:**

This was a retrospective, cross-sectional study which included endurance runners (*N* = 710; 51.5% female; 35.4 ± 15.5 years; 51.6% were training competitively at the time of testing) with different running injury histories. Runners self-reported foot strike pattern [rearfoot, non-rearfoot (mid or forefoot), or “don't know”] and information about shoewear specifics. All runners performed a single session of running at self-selected speed on an instrumented treadmill with 3D motion capture and high-speed filming that verified actual foot strike. Logistic regression was used to predict accuracy of foot strike detection and RRI.

**Results:**

Overall accuracy of foot strike detection was low (42.7%; *p* < 0.01). Self-reported foot strike was 28.3% for rearfoot, 47.0% for nonrearfoot forefoot strike and 24.6% did not know. Biomechanical analyses actually showed that 34% of rearfoot strikers accurately detected rearfoot strike, while 69.5% of non-rearfoot strikers self-reported accurate non-rearfoot strike (*p* < 0.05). Runners who “did not know” their strike had the highest prevalence of RRI compared to runners who self-reported nonrearfoot or rearfoot strike (73% vs. 56% and 58%; *p* < .001). After accounting for several variables, shoe heel-to-toe drop was a consistent predictor of accurate strike detection [OR = 0.93 (0.88–0.99); *p* = 0.026] and RRI in last six months [OR = 1. 1 (1.01–1.17); *p* = 0.018]. RRI were also predicted by recent shoe change [OR = 2.8 (1.7–4.6); *p* < 0.001].

**Discussion:**

Accurate detection of actual foot strike by endurance runners varies by the actual foot strike type determined during testing and is associated shoe characteristics. These findings demonstrate the importance of accurately identifying foot strike pattern and recommending footwear as a factor if planning to use retraining to alter foot strike pattern.

## 1 Introduction

Foot strike patterns during running have garnered considerable attention over the last 15 years as potential contributors to running related injury (RRI) and economy. In general, rearfoot strike pattern occurs when initial foot contact is made on the heel or rear one-third of the foot, whereas nonrearfoot striking involves initial contact on the front half of the foot that may be followed by heel contact (1). Among recreational and competitive runners, the predominant foot strike type is rearfoot, with lower prevalence in the nonrearfoot or mixed strike patterns (2–4). It has been suggested that foot strike pattern affects the mechanical properties and functioning of the lower extremity joints such as the ankle, knee and hip, and soft tissue complexes such as the gastrocnemius-Achilles tendon (5–7). Low quality evidence also suggests that foot strike pattern may be associated with running-related injuries (RRI) but this relationship is still evolving (8). Foot strike pattern has the potential to influence how forces are absorbed and disseminated along the kinetic chain. Both rearfoot strike and forefoot strike may be related to specific injuries depending on the study (9). The combination of foot strike pattern and loading responses to repetitive collisions with the ground (ground reaction force, load rate) (10, 11), coupled with kinematic features of running (12, 13) can stimulate beneficial or deleterious changes in soft tissue and bone depending on the mechanical and force-generating factors. Cross sectional and prospective evidence suggests that RRI prevalence is high, ranging from 49% to 92% depending on the study (14–17). Further, it has been reported that rearfoot strikers have running-related repetitive injury prevalence that is twice that of forefoot strikers, and lower variability of foot contact angle which may amplify localized tissue loading RRI risk (18).

One common practice to address RRI as part of a gait retraining approach is to adjust foot strike and potentially modify stresses along the kinetic chain (6, 19–21). Typically, foot strike change involves shifting from rearfoot to non-rearfoot pattern in an attempt to mitigate vertical loading rates, to offload mechanical stresses along the lower extremity, to prepare for transition to minimalist shoes, or to improve contraction of posterior leg muscles and mechanical efficiency (6, 19, 20, 22). The strategy of promoting a non-rearfoot strike pattern is founded on the potential of improving a number of mechanical parameters such as tibial acceleration, patellofemoral stress, ankle joint stiffness, average vertical loading rate, and peak loading rate (22–25). However, in addition to the possibility that there are beneficial effects to adopting a nonrearfoot strike pattern, whether such a strategy can in fact be part of gait retraining is dependent on whether runners are aware of their running pattern before they attempt to modify their pattern. Our anecdotal observations of over two thousand runners in our running medicine program have revealed that runners who have not purposefully attended to their gait form, have never had form evaluated and who have never seen themselves run are not often accurate with estimating how they interact with the ground. This observation, important to both patients and clinicians, contributed in part to the development of this research question that addresses a large gap in the literature: determining the relationship between foot strike detection accuracy and the prevalence of RRI.

Extrinsic factors, such as running shoe characteristics, can modulate the interaction of the foot with the ground and thereby influencing foot strike pattern (26, 27). Shoe features such as weight, heel height, midsole cushion and heel-to-toe drop and material properties can affect lower limb posture and dynamics, particularly at initial collision, or by altering stance time (28, 29). Shoe selection has been associated with foot strike pattern in some investigations, where “minimal” style of shoes promote a more non-rearfoot initial contact than traditional running shoes, which promote more ankle dorsiflexion and knee flexion at initial contact (27, 30). Systematic reviews indicate that increased shoe sole thickness may also decrease plantar sensation (31) which can make it more challenging for runners to be aware of, or adjust foot strike pattern due to attenuation of afferent feedback during loading.

The primary purpose of this study was to determine the accuracy of self-reported foot strike pattern among endurance runners, and the secondary purpose was to identify what individual and shoe wear factors were predictive of accurate foot strike detection and recent RRI. It was hypothesized that (1) the overall foot strike detection accuracy would be low, particularly among rearfoot striking runners who wear shoes with high heel height or relatively high heel-to-toe drop, and (2) runners who did not accurately detect foot strike pattern would report higher prevalence of RRI than accurate detectors.

## 2 Materials and methods

### 2.1 Study design

**Figure 1:**
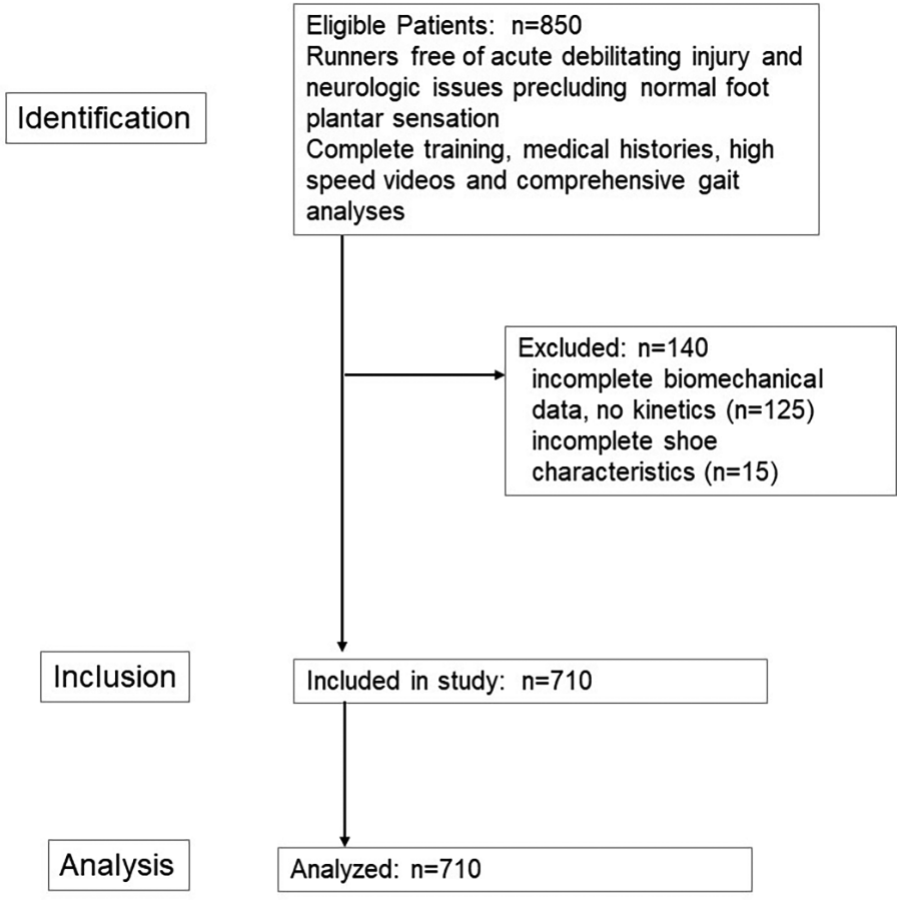
STROBE study flow diagram for observational studies.

The study design was a retrospective cross-sectional. This study and all its procedures followed the guidelines for the Declaration of Helsinki's Protection of Human Subjects and was approved by the University of Florida Institutional Review Board (#202401340). The recommended format for observational studies, described by the statement in strengthening the reporting of observational studies in epidemiology (STROBE), is used here (32). The Study Flow Diagram is shown in Figure 1.

### 2.2 Setting

The Exercise and Functional Fitness Laboratory is located in a quaternary health care facility. Data from all runners who obtained running analyses for performance and injury prevention services were pulled for analysis from January 2016 to December 2022.

### 2.3 Participants

Of all the runners who sought clinical gait services during this time (*N* = 850) a total of 710 were eligible and had complete data available for analysis. All runners provided written informed consent to allow their data to be stored in our institutional research databank. Inclusion criteria were: aged 12–77 years (spanning from middle school cross country to older master runner), all races and ethnicities, both sexes and different levels of experience. Exclusion criteria were: (1) the presence of any current (at the time of testing) or recent (during the 6 months prior to testing) traumatic debilitating musculoskeletal injury (ankle sprain, hamstring strain, stress fracture or Anterior cruciate ligament rupture); (2) Pre-existing chronic neurological conditions that interfere with normal plantar surface sensitivity or proprioception (conditions such as peripheral neuropathies or previous traumatic damage to nerves innervating the lower extremities). If any runner had a history of a stress fracture related to running, a minimum of 6 months was required to ensure the runner has been medically cleared and was returning to training. All runners had been training to run in a competitive event for running which was a minimum of 20 km per week with the event being a minimum 5 km distance. Minimum average weekly program typical running distance of this population sample was 33.2 ± 2.6 km.

#### 2.3.1 Characteristics

Runner characteristics included the following: demographic and anthropometric characteristics, run training history (volume, type, years of experience), shoe wear, and current training status for competition (yes, no). The make and model of running shoe was collected from each participant and specifications included heel height (cm), heel-to-toe drop (cm) and shoe weight (oz for standard size 9, 10 women's and men's, respectively) (33). Injuries were defined as being the underlying cause of musculoskeletal pain which limited or led to the cessation of running (distance, velocity, duration, or training) for at least 7 days, or interfered with at least three consecutive training sessions, or led to the participants having to consult a physician about the symptoms related to running (34). Information about RRI was collected from each participant including location and type of pain, and whether or not they had experienced any previous RRI within the last 6 months prior to testing. Injuries were classified as either a bony or soft tissue injury.

Based on recommendations to identify injury risks (33), additional questions included: (1) have you purposely tried to change your foot strike over the last 6 months? (yes, no); (2) have you changed your running shoe wear type over the last 6 months? (yes, no); and (3) do you use any additional insert(s) in your shoe? (yes, no). Runners were asked to choose what type of foot strike they perceived they adopt when running. Choices included “rearfoot”, “non-rearfoot” (“mid-foot” or “forefoot”) or “don't know”.

### 2.4 Instrumentation and data collection

A standard analytic process was performed and select variables were presented at foot strike during the gait cycle, such as sagittal joint angles (35, 36). Motion capture during running at self-selected speed was captured using a high-speed, seven-camera 3D optical motion analysis system (Motion Analysis Corp, Santa Rosa, CA, USA) that sampled at 200 Hz and was synchronized with force plate data collected from an instrumented treadmill (AMTI, Watertown, MA; USA) at 1,200 Hz.

#### 2.4.1 Testing procedure

Retroflective markers were affixed bilaterally over the acromion processes of the shoulders, mid-distance between the acromion and elbow lateral epicondyles (tricep muscle belly), lateral epicondyle of the elbow, mid-distance between lateral epicondyle of elbow and radial styloid process on posterior surface of forearm, mid-distance between styloid processes of radius and ulnar (dorsal wrist), posterior superior iliac spine, anterior superior iliac spine, mid-distance between ASIS and patella (anterior thigh), medial and lateral condyles of the femur, tibial tuberosity, medial and lateral malleoli, calcaneus, lateral to the head of the fifth metatarsal, and medial to the base of the hallux (37). One offset marker was placed on the right scapular inferior angle. Prior to data collection, a static calibration trial was performed to generate the computer model of the runner in the software (Cortex, Motion Analysis Corp, Santa Rosa, CA, USA). The medial knee and ankle markers were removed for all running conditions to prevent the markers from being accidentally knocked off the skin during the testing.

Participants ran for 8 min during an acclimation period at a pace defined as a “pace used for long run distance training”. Kinematic and kinetic data were obtained at this time to ensure that each runner's gait had stabilized. Between minute 9 and 10, a 10-s sample of data was recorded (average of 12–14 strides). Slow-motion videos were also captured for reference in the sagittal and frontal planes (Casio Elixim; Casio America, Inc., Dover, NJ. USA).

#### 2.4.2 Data processing

**Figure 2:**
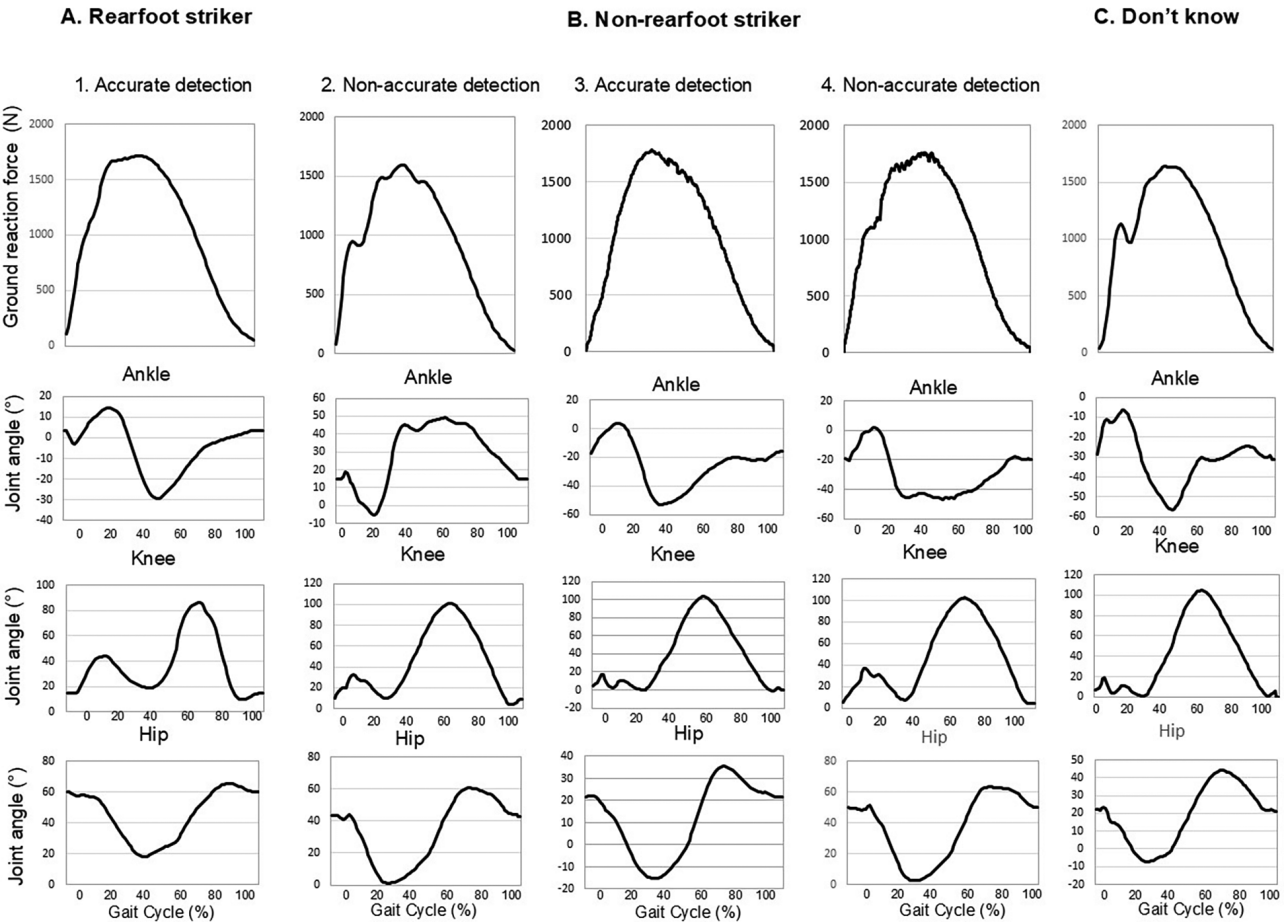
Sample ground reaction force (GRF) curves shown for a runner in each of the five groups during stance **(A1)** Rearfoot accurate, **(A2)** Rearfoot non-accurate, **(B3)** Non-rearfoot accurate, **(B4)** Non-rearfoot non-accurate and **(C)** Don't know.) GRF are expressed in N. The serial panels beneath each GRF trace are joint motion curves during an entire gait cycle. GRF and motion values were sampled at 100 Hz.

Marker data were filtered at 9 Hz with a fourth-order, low-pass Butterworth filter. Bone models were created for every runner with an individual center of mass (COM) location using the methods of de Leva et al. (38) using commercially-available software (Visual3D, C-Motion, Inc; Germantown, MD). A complete gait cycle was defined at the commencing with initial foot contact (0%) and completed at the subsequent ground contact of the same foot (100%). A 20N threshold was used to set the initial foot contact and toe-off events (36). Joint angles at initial contact for the ankle, knee, hip and pelvis were also determined (traces provided in Figure 2). Based on the foot markers, the ankle angle at foot strike was calculated as the angle between the foot segment and the ground at initial contact relative to the standing angle. The reference point for joint angles was established at 90° as vertical for the knee and hip and 0° as horizontal for the ankle angle. The pelvis was developed from the anterior and posterior superior iliac spine markers, and the anterior inclination was expressed relative to the horizontal as 0°of anterior tilt (35, 36).

Temporospatial characteristics including cadence (steps/min^−1^) and the vertical displacement of the COM (the difference between the minimal and maximal vertical height of the COM during a gait cycle), and stride width (the medial-lateral distance between the proximal end position of the foot at foot strike to the proximal end position of the foot at the next contralateral foot strike) were calculated. Stance time was determined as the time (in seconds) that each foot was in contact with the treadmill. All of the temporospatial data and biomechanical parameters above are provided to demonstrate the consistency of performance of this runner cohort with other published studies. These variables were calculated using commercially available software (Visual3D, C-Motion, Inc; Germantown, MD and MatLab, Mathorks; Natick MA; USA). Bone models were developed for each runner with the individual COM location using the method described by de Leva (38). The marker tracking data from the bone models were used to calculate the temporal spatial parameters and joint angles. Right and left side values for these measures were averaged and reported in the results.

Force data were collected from the instrumented treadmill at a frequency of 1,200 Hz. GRF data were low pass filtered at frequency cutoff of 40 Hz using a 4th order Butterworth filter. The peak vertical component of the peak ground reaction force (GRF). Vertical average loading rate (VALR) was calculated as the angle of the slope for the force-time signal the slope of the ΔF/Δt of the most linear portion of the force curve, where ΔF is the change of vertical force and *Δ*t is the change of time between 20%–80% of the first rise to the initial peak of the vertical GRF (39). When no initial peak was present based on computer-generated tracings, VALR was defined as vertical GRF at 13% of stance (40). Vertical leg stiffness was estimated using the following: K_vert_ = F_max_/Δy, where F_max_ was the peak vertical force and Δy was the maximum vertical displacement of the COM (41) that occurred during the entire gait cycle.

Actual foot strike type pattern for each participant was determined by the initial contact point with the treadmill, using the initial contact angle between the foot segment and the horizontal ground at foot initial contact. A single investigator (HKV) reviewed all the sagittal view of high-speed reference video recordings obtained at 300 fps using a high-speed camera (Casio Elixim; Casio America, Inc., Dover, NJ. USA) and visually confirmed foot strike pattern (9). Rearfoot strike pattern was defined as when the initial point of contact was by the confirmed if the point of initial contact was on the heel or on the rear one third of the plantar surface of the foot, and non-rearfoot strike was defined as when the point of contact at confirmed if the initial contact point was on the front two thirds of the plantar surface of the foot (1).

#### 2.4.3 Runner categorization

Based on the combination of foot strike and accuracy of foot strike detection, runners were then placed into five categories for statistical analysis: *Rearfoot accurate, Rearfoot non-accurate, Non-rearfoot accurate, Non-rearfoot non-accurate* and *Don't know*.

### 2.5 Statistical considerations

Statistical analyses were performed using SPSS version 29.0 (IBM, Armonk NY). Normality of the data (skewness, kurtosis) was confirmed using Shapiro-Wilk tests, and descriptive statistics were calculated for all variables and demographic characteristics. Analyses of covariance (ANCOVA) were performed on the five runner groups to test for differences in demographic, anthropometric and training history continuous variables. Covariates were age and running velocity. Chi-square tests (*χ*^2^) were used to determine if there were group differences in categorical variables (demographics, accuracy of foot strike type, RRI by detection accuracy). ANCOVAs were used to test group differences for biomechanical variables, where the between-group factor was foot strike accuracy (Rearfoot accurate, Rearfoot non-accurate, Non-rearfoot accurate, Non-rearfoot non-accurate and Don't know). Covariates were age and running velocity. If significant group differences were detected, Tukey *post hoc* tests were used to determine where group differences existed. For non-parametric and parametric tests, significance was established at *p* < 0.05. The eta squared (*η*^2^) were provided to show the effect sizes for continuous variables; values of 0.01, 0.06 and 0.14 represented negligible to small, medium and large effects (42).

Logistic regression was used to determine the risk factors which may predict accurate detection of foot strike pattern and history of RRI in the last year. The choice of factors to be entered into the model were based on previous work showing age and BMI affected running biomechanics (36, 43), and on the potential impact of the running shoe characteristics. As such, factors first entered into the model were age and BMI, followed by shoe characteristics (heel height, heel-to-toe drop, weight), responses to ‘*Have you tried to change foot strike type*? and ‘*Have you changed running shoes in the last 6 months*?’, and running speed. Odds risk (OR) values were obtained for each variable entered into the regression models. *a priori* alpha levels were established at 0.05 for all statistical tests.

## 3 Results

### 3.1 Runner and shoe characteristics

The key demographics were similar among the five groups of runners based on accuracy of self-reported foot strike pattern (Table 1). The weekly distance, however, was lowest in the non-rearfoot non-accurate group (*p* = 0.001). The non-rearfoot, accurate strikers used shoe inserts least frequently (*p* = .005), and wore shoes that had, on average, a lighter weight, lower heel-to-toe drop, and lower heel height than the other groups (all *p* < 0.05). Fewer runners in the non-rearfoot, accurate group had the lowest prevalence of RRI (*p* = 0.001). The effect sizes were all considered small.

**Table 1:** Characteristics of runners categorized by self-reported foot strike pattern, irrespective of accuracy (*N* = 710).

Actual strike	Rearfoot		Non-rearfoot			
Accurate detection	Yes (*n* = 182)	No (*n* = 212)	Yes (*n* = 121)	No (*n* = 18)	Don't know (*n* = 141)	*p η* ^2^
Age (y)	35.8 ± 15.4	35.6 ± 15.1	35.2 ± 13.8	43.4 ± 17.8	34.2 ± 16.7	0.188.009
Sex, female (%)	52.7	50.7	50.4	52.6	51.4	0.969 —
Race (%)						
White, Caucasian	87.4	89.2	86.0	94.7	83.4	—
Black, African American	5.5	3.8	6.6	5.3	2.9	—
Other	7.1	7.0	7.4	0	13.7	0.333 —
Ethnicity, Hispanic (%)	0.5	1.4	0.8	0.0	2.9	0.331 —
Height (cm)	171 ± 11	170 ± 10	172 ± 9	172 ± 6	169 ± 10	0.271.007
Weight (kg)	67.4 ± 15.5	66.7 ± 13.3	67.1 ± 11.8	66.1 ± 7.8	65.8 ± 15.1	0.858.002
BMI (kg/m^2^)	22.8 ± 3.7	22.7 ± 3.2	22.6 ± 3.2	22.4 ± 1.6	22.6 ± 3.4	0.975.001
Running history						
Years (#)	10.4 ± 11.1	9.9 ± 10.3	10.4 ± 10.1	14.3 ± 15.0	10.3 ± 12.1	0.643.004
Weekly distance (km)	31.7 ± 21.12	36.2 ± 32.2	40.6 ± 29.3	27.8 ± 20.6^a^	28.0 ± 21.12^a^	0.001.026
Runs week (#)	4.0 ± 1.9	4.0 ± 1.5	4.1 ± 1.7	3.4 ± 1.5	3.7 ± 2.2	0.239.008
Preparing for a race (%)	45.2	54.6	60.2	55.6	44.8	0.149 —
Tried to change foot strike in last 6 months (%)	27.2	26,2	27.0	36.8	16.0	0.062 —-
Use shoe insert (%)	23.6	15.1	9.1	27.8	22.7	0.005 —
Shoe characteristics (%)						
Heel height (cm)	31.1 ± 5.9^b^	30.2 ± 6.3	28.2 ± 6.6	28.2 ± 8.4	31.0 ± 6.8^b^	0.004.024
Heel-to-toe drop (cm)	8.4 ± 3.2^b^	7.7 ± 3.5^b^	6.0 ± 3.6	7.4 ± 4.2	8.1 ± 3.4^b^	<.001.054
Weight (oz)	9.4 ± 1.7 ^b^	9.0 ± 1.5	8.5 ± 1.3	8.5 ± 1.3	9.1 ± 1.5	<.001.037
Shoe use (km)	185 ± 168	163 ± 136	154 ± 161	150 ± 99	136 ± 141	0.116.013
History of RRI (% yes)	59.9	57.7	42.1	52.1	73.0	0.001 —
Bony injury (% yes)	26.0	23.9	10.5	26.4	34.1	0.086 —

BMI, body mass index; RRI, running related injury.

^a^ = Different than Rearfoot incorrectly detecting strike and Non-rearfoot correctly detecting strike at *p* < 0.05.

^b^ = Different than Non-rearfoot correctly detecting strike at *p* < 0.05.

Values are means ± SD or% of the group.

### 3.2 Foot strike detection accuracy

**Figure 3:**
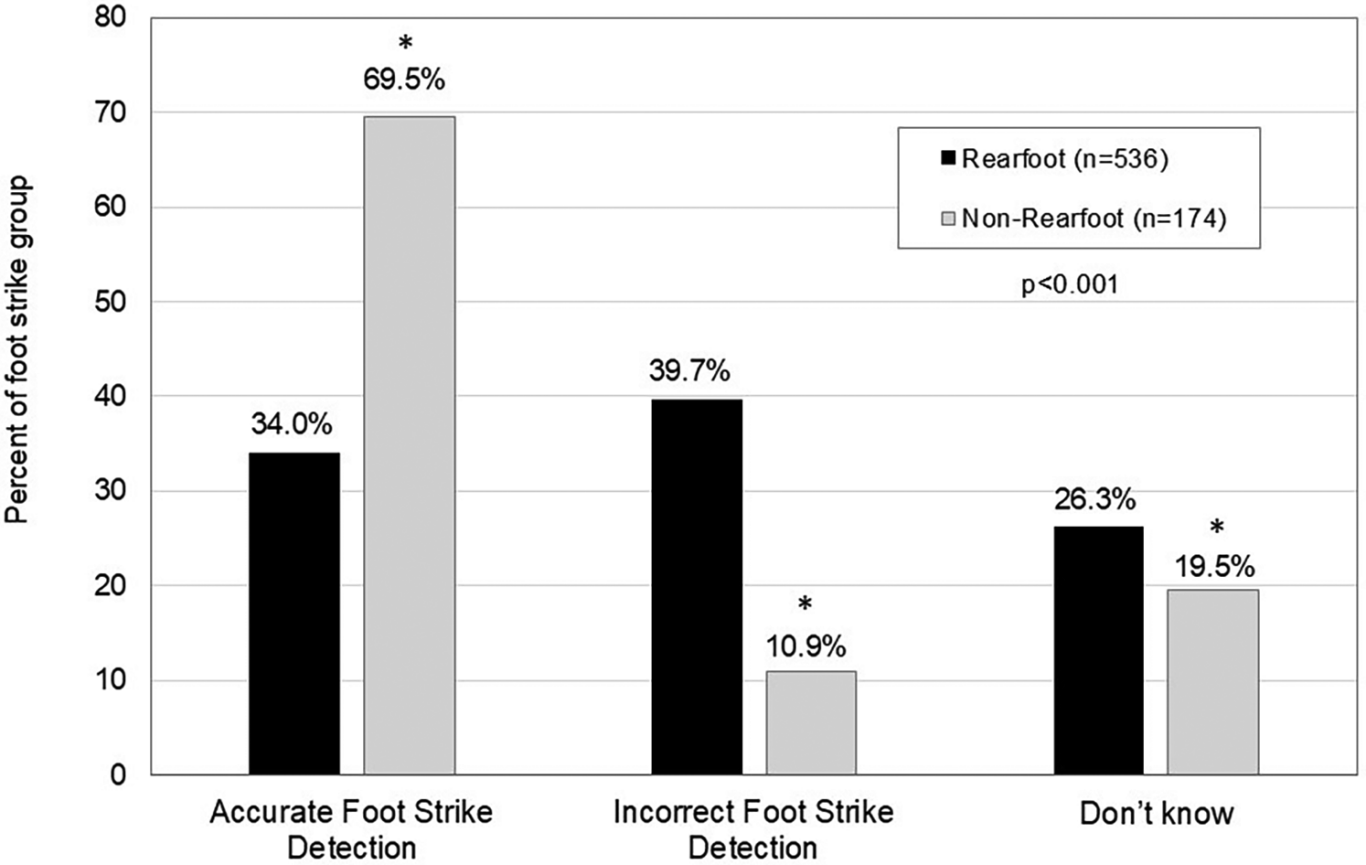
Accurate self-report detection of habitual foot strike type. Values are expressed as% correct based on actual foot strike pattern. *denotes different than rearfoot strikers at *p* < 0.05.

Overall, of the 710 runners, 28% and 47% of runners self-reported their foot strike patterns as rear foot and non-rearfoot, respectively, with 25% indicating they did not know what their pattern was. The actual foot strike distribution was that 76% of runners were rearfoot strikers and 25% were non-rearfoot strikers (Figure 3). Only 34% of rearfoot strikers accurately detected foot strike pattern, however, 70% of non-rearfoot strikers detected actual foot strike accurately. Of the runners who did not know their strike pattern, 81% were actually rearfoot striking and 19% were non-rearfoot striking.

#### 3.2.1 Key biomechanical variables

Table 2 provides key biomechanical variables by accuracy of self-reported foot strike patterns. The mean running velocity was 2.7 ± 0.5 m/s, with no difference among groups (range 2.7–2.8 m/s; *p* = 0.308). In the non-rearfoot strikers, irrespective of accuracy, mean ankle joint angles were lower (more plantar flexed position) and knee joint angles were lower (more flexed knee position) at initial foot contact compared to the other three groups (both *p* < 0.001). Cadence and stance time were different in the non-rearfoot accurate runners than rearfoot accurate runners and those who didn't know their strike (*p* = 0.001). Peak vertical GRF values were higher in the non-rearfoot accurate runners compared to rearfoot runners irrespective of accuracy (*p* < 0.05). Effect sizes were considered medium for ankle angle at foot strike and large for VALR. Figure 2 provides the sample joint motion traces for the ankle, knee, and hip for each of the five study groups during a typical gait cycle.

**Table 2:** Key mechanical variables of running by accuracy of self-reported foot strike pattern. Biomechanical values are the average of right and left sides and are expressed as means ± SD.

Actual strike	Rearfoot		Non-rearfoot			
Accurate detection	Yes	No	Yes	No	Don't know	*η* ^2^
Foot strike joint angle, sagittal (°)						
Ankle*	69.9 ± 5.8	69.2 ± 5.4	59.5 ± 7.1^a,b,e^	60.2 ± 6.5^a,b,e^	68.5 ± 8.2	0.259
Knee*	78.6 ± 4.3	78.6 ± 4.3	76.7 ± 4.7^a,b,e^	76.0 ± 3.8^a,b,e^	78.6 ± 4.8	0.028
Hip	35.8 ± 6.5	34.9 ± 7.0	36.0 ± 6.6	35.1 ± 6.4	35.7 ± 6.9	0.006
Pelvis	17.0 ± 5.1	16.2 ± 6.1	17.5 ± 5.1	16.5 ± 4.9	17.3 ± 5.3	0.008
Cadence* (step/min)	166 ± 10	168 ± 10	172 ± 10 ^a,b,e^	173 ± 14	167 ± 11	0.020
Stance time (s)*	0.27 ± 0.03	0.26 ± 0.03	0.25 ± 0.03^a,b,e^	0.24 ± 0.02	0.27 ± 0.03	0.048
Step length (m)	0.93 ± 0.19	0.93 ± 0.15	0.95 ± 0.15	0.91 ± 0.20	0.91 ± 0.20	0.008
Stride width (cm)*	8.3 ± 2.6	8.5 ± 2.4	9.6 ± 3.9^a^	9.1 ± 2.9	8.7 ± 5.4	0.018
Peak vGRF (BW)*	2.3 ± 0.3	2.4 ± 0.4	2.5 ± 0.3^a,b^	2.4 ± 0.3	2.4 ± 0.3	0.032
VALR (BW/s)*	67.1 ± 21.3	69.1 ± 21.0	50.7 ± 17.4 ^a,b,e^	50.9 ± 18.5^a,b,e^	65.2 ± 22.1^c^	0.130
K_vert_ (*N*/cm)	174.4 ± 38.4	176.2 ± 35.4	174.3 ± 27.3	180.4 ± 36.9	168.9 ± 39.3^d^	0.008

vGRF, vertical ground reaction force; BW, body weight; K_vert_, vertical leg stiffness; VALR,s vertical average loading rate.

^a^ = Different than Rearfoot group, accurate at *p* < 0.05.

^b^ = Different than Rearfoot group, not accurate at *p* < 0.05.

^c^ = Different than Non-rearfoot group, accurate, at *p* < 0.05.

^d^ = Different than Non-rearfoot group, not accurate, at *p* < 0.05.

^e^ = Different than Don't know at *p* < 0.05.

* denotes group difference at *p* < 0.05.

#### 3.2.2 Running related injury history and foot strike pattern

**Figure 4:**
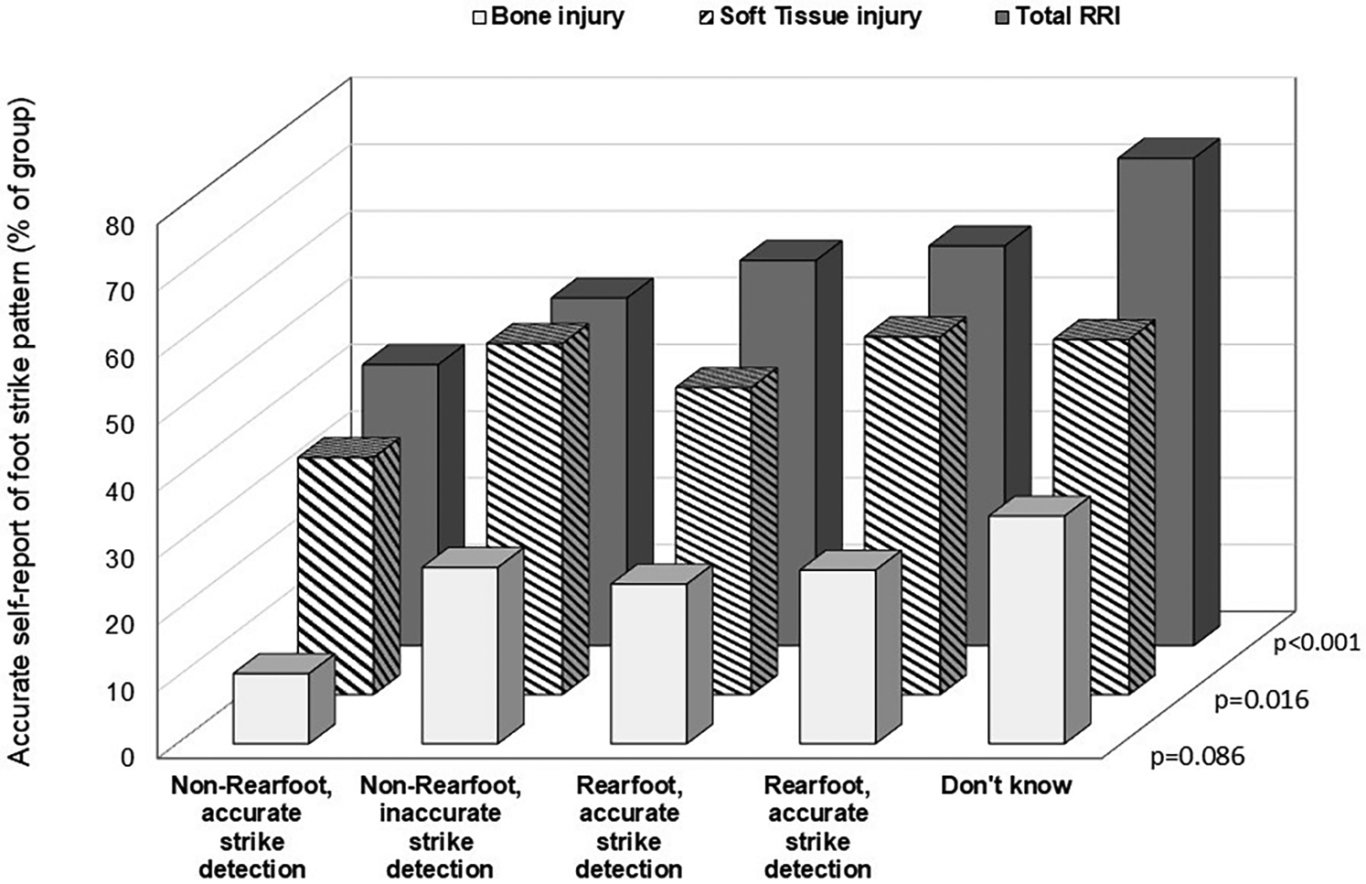
Prevalence of reported running-related injuries (RRI) by accuracy of self-report foot strike detection. Values are shown as percent of the group for total RRI, soft tissue injuries and bone injuries. Soft tissue injuries include: Achilles tendinopathy, Iliotibial band syndrome, hamstring strain pain, patellofemoral pain, plantar foot pain, flexor hallucis longus tendinopathy, posterior tibialis tendinopathy, low back pain, and Hoffa's fat pad inflammation. Bone injuries included: medial tibial stress syndrome, stress fractures of the tibia, fibula, metatarsals, cuneiforms, navicular, femur, and pelvis.

The overall prevalence of RRI in the last 6 months was greatest among the runners who didn't know their habitual foot strike and lowest among the non-rearfoot accurate strikers (Figure 4; *p* < 0.001). The proportion of runners who reported soft tissue injuries were 11%–18% less among the non-rearfoot accurate runners compared to rearfoot strikers (*p* = 0.016).

#### 3.2.3 Regression analyses

After accounting for age, BMI, running speed, and years of experience, purposefully trying to change foot strike type (yes) was associated with an increased OR for accurately detecting actual foot strike [OR = 1.51 (95% CI = 1.010, 2.352); *p* = 0.045](Table 3A). Higher heel-to-toe drop values were associated with lower likelihood of accurate foot strike detection [OR = 0.931 (95% CI 0.875–0.991); *p* = 0.026] (Table 3A). Changing shoe types over the previous 6 months and higher heel-to-toe drop values were both associated with having a RRI (OR = 2.79 and OR = 1.09, respectively; *p* < 0.05).

**Table 3:** Regression models to predict accurate detect foot strike pattern (3A) and predict history of running related injury (RRI) in the last six months (3B).

A. Accurate detection of foot strike pattern		
Predictor	Odds ratio [95% CI]	*p*
Age (y)	1.002 [0.987–1.017]	0.807
BMI (kg/m^2^)	1.032 [0.976–1.092]	0.269
Running Speed (m/s)	1.135 [0.953–1.351]	0.156
Running experience (y)	0.993 [0.974–1.012]	0.456
Tried to change foot strike type (yes, no)	1.541 [1.010–2.352]	0.045
Changed shoe type in last 6 months (yes, no)	0.725 [0.478–1.100]	0.130
Shoe heel height (cm)	1.009 [0.978–1.041]	0.578
Shoe heel-to-toe drop (cm)	0.931 [0.875–0.991]	0.026
Shoe weight (oz)	1.149 [1.000–1.320]	0.050
B. Running Related Injury in the past six months		
Predictor	Odds ratio [95% CI]	*p*
Age (y)	1.004 [0.987–1.021]	0.623
BMI (kg/m^2^)	0.947 [0.890–1.007]	0.083
Running Speed (m/s)	0.836 [0.690–1.015]	0.070
Running experience (y)	1.018 [0.995–1.041]	0.126
Tried to change foot strike type (yes, no)	0.892 [0.552–1.441]	0.639
Changed shoe type in last 6 months (yes, no)	2.785 [1.678–4.621]	<.001
Shoe heel height (cm)	0.993 [0.959–1.028]	0.678
Shoe heel-to-toe drop (cm)	1.087 [1.014–1.165]	0.018
Shoe weight (oz)	0.886 [0.759–1.033]	0.126

BMI, body mass index.

## 4 Discussion

A key finding of this analysis was that rearfoot striking runners were the least likely to be able to accurately detect foot strike patterns, especially when shoes had a higher heel-to-toe drop or greater weight. Non-rearfoot strikers with accurate detection of strike had the lowest prevalence of total and soft tissue RRI compared to the other groups. Factors that were contributors to accurate detection of foot strike patterns included higher shoe heel-to-toe drop, greater shoe weight and purposefully trying to change foot strike type over the previous months. Recent RRI were predicted by changing shoe type over the last 6 months and shoe heel-to-toe drop. Our hypotheses were supported in part by these findings. Awareness of foot strike pattern, when coupled with running shoes of lower weight and drop, may reduce the likelihood of an RRI potentially due to better sensory input from the ground, which can help alter foot strike pattern and limb mechanics.

A previous study examined runners (*N* = 60) who had been formally trained in being able to change running technique and showed there was an overall higher accuracy of strike detection at 68.3% (rearfoot strikers in traditional shoes were correct 90.9% of time, and anterior foot strikers were correct 58% of the time) compared to the current study (30). The technique-based training in this earlier study included more runners formally trained in specific methods such as Chi or Pose, which may improve awareness of foot contact patterns. In contrast, related studies show that recreational runners also have poor knowledge of foot arch type and dynamic pronation, as only 48.9% were able to correctly identify arch pattern and 10% correctly identified overpronation (44).

A recent systematic review with and meta-analysis reported that foot strike pattern was closely related to ankle angle at initial contact, low ankle dorsiflexion, negative work at the ankle and knee, vertical stiffness and load rate (7). We found that greater plantarflexion and knee flexion angle occurred at initial foot contact with corresponding lower VALR among non-rearfoot strikers. Further, leg stiffness was lowest among runners who did not know how they struck the ground. Stride width was highest among non-rearfoot runners who correctly self-reported foot strike pattern. The current findings are in agreement with systematic review which reported that sagittal plane kinematics differ slightly between runners of either forefoot or rearfoot strike patterns and load rates (3). Further, rearfoot strikers demonstrated higher vertical stiffness which is consistent with the observation that impact loading can be modulated by greater vertical compliance, which can be achieved in part through ankle plantarflexion and knee flexion (7, 45). Ankle dorsiflexion may facilitate eccentric muscle actions of the anterior tibialis (45), and knee flexion excursion during early stance (46), which may be related to energy absorption and modulation of stiffness. A few possibilities exist to explain our other findings above. First, some runners in each group who had recovered from recent RRI were transitioning in real-time to a new foot strike pattern to reduce injury risk and may not have yet established a consistent running pattern. Alternatively, runners may alter gait characteristics including reducing ankle dorsiflexion to help offset the pain (47), which can bilaterally alter features of gait (48). Second, with different RRI histories and acuity of injury, some runners in each group may have had residual or asymmetric gait mechanical variability related to the previous injury type (49). Further, current or recent injuries are related to higher variability in foot strike index, knee-ankle-foot movement coupling, and lower trunk-pelvis coupling; 73% of injured runners have mechanical variability in at least one mechanical variable (49). Thus, even if the foot strike pattern was self-reported accurately, mean values of mechanical characteristics may contain the variability from injuries that washed out statistical significance.

Misjudging strike pattern has implications for successful adoption of retraining programs, and may influence shoe selection (44). Specifically, runners who can more accurately detect foot strike pattern are likely going to be successful with responding to therapy cues and modifying foot strike pattern compared to runners who lack this ability. One study found that runners who have poor understanding of foot strike and foot mechanics or who have pain or discomfort may choose heavier shoes with more cushioning and thicker heels to reduce discomfort (44). A change in footwear may lead to reduction in pain, however heavier shoes and different heel-to-toe drop may affect foot strike pattern which is associated with RRI. Conversely, runners who wish to transition from shoes with high cushioning or energy absorptive properties compared to minimalist shoes may enhance RRI risk due to the challenge of managing impact loading through the musculoskeletal system which has not adapted to a less absorptive shoe (30). It has been shown that highly cushioned maximalist shoes induce 7%–14% greater VALR and 6%–12% higher impact loading peaks compared to traditional running shoes especially at faster speeds (50). Further, stance time is prolonged with use of shoes with thick midsoles compared to thinner soles (28), a trait that may challenge adoption of faster turnover with gait retraining.

Shoe characteristics are related to foot strike pattern (11, 27, 51). Progressively increasing heel-to-toe drop acutely in the laboratory from 0 mm to 8 mm induced a reduction in knee flexion angle and increased ankle dorsiflexion angle at initial contact during overground running, ankle excursion in the sagittal plane, and reduced loading rates, while increasing load rates while treadmill running (52). In a large prospective, intervention study of runners (only a quarter of whom had experience with low drop shoes) who were provided shoes of different heel-to-toe drop heights, it was found that “low drop” shoes of ≤6 mm were associated with a 52% lower hazard ratio for RRI among occasional novice runners and a 67% elevated risk for RRI among regularly trained runners over 6 months (53) The main conclusions of these papers were that acute reduction of hoe drop was related to lower treadmill loading rates (52), and that the long-term effect of shoe drop was “neutral” on incidence of RRI (53). These previous studies included runners with widely varied running experience and weekly training levels, and categorized experience with “low drop” shoes as being less than <10 mm, which was greater in the current study (53). Differences in definitions in shoe wear, coupled with variable testing surfaces and different training levels likely contributed to divergent findings in these previous studies.

A potential interpretation of the current findings could be that positioning a foot in a shoe with an elevated heel contributes to the sensation of being front loaded on the plantar surface (mid-to-forefoot), which in turn may create the sensation that the runner is already running on the forefoot. This sensation may be enhanced with customized or off-the-shelf-shoe inserts, as excessive material between the plantar surface and the ground can compromise sensation (29). Rearfoot strikers who are encouraged to land without the heel contacting the ground during retraining may find it difficult to sense when they are actually achieving this therapeutic goal. In contrast, habitual non-rearfoot strikers who wear lighter shoes with less drop may have better sensory input from the ground, which in turn can help modify foot strike pattern and subsequently, limb mechanics. It has been proposed that these modifications in stiffness and loading are achieved through a motor learning effect, or a “central control strategy”, that promotes increased stiffness modulation at the knee and less stiffness at the ankle (54). Gait retraining involves repeated practice with cues and feedback on specific techniques including faster cadence and shorter stance time, with soft, non-rearfoot strikes to possibly dampen impact loading and ground reaction forces (55–57). Whether or not success of foot strike modification persists with different types of shoewear has yet to be determined. What is known is that runners respond to shoes with less heel-to-toe drop by shortening stance time, slower shank retraction velocity, and greater vertical stiffness compared to heavier shoes with more drop (29). These responses reflect altered movement control with loading which can influence load attenuation during running. Moreover, afferent feedback may be processed more quickly after foot strike, leading to shorter stance and faster cadence. There is variability in how runners respond and develop movement strategies to gait retraining cues (58). Different types of shoe modifications may facilitate proprioception and foot strike change for runners who need different types of sensorial input.

### 4.1 Limitations, strengths and future directions

This was a cross-sectional study, and to establish causation between foot strike pattern and RRI, a longitudinal design would be necessary. This retrospective study was limited by the factors which had been measured at the time of data collection, but did not include other characteristics which may influence, or be indicative of, foot strike pattern such as: foot plantar pressures, and that may impact the perception of foot strike, are arch type or shape (59), actual contact area of the foot in the shoe (60), shoe materials, parallel use of multiple shoes (61), and individual degree of wear based on mileage used (62). This study was able to use logistic regression for multiple factors across a large sample. Therefore, the findings may be generalized to the greater running population across the lifespan.

The findings from this study provide the foundation for exploring the need to modify foot strike pattern and if this is possible as part of routine gait retraining. Future work can include computer modeling methods to identify likely muscle activation changes and biomechanical outcomes in relation to foot strike patterns and shoe characteristics. Incorporation of electromyogram measures concurrent with motion testing would add important insight into neuromotor functions of running with different shoe wear and RRI and discriminate responsiveness to gait retraining. Prospective studies will provide additional clarity as to whether foot strike pattern and type of shoe are related to the development of specific RRI.

### 4.2 Conclusion

Accurate foot strike detection is challenging for endurance runners across the age span. Contributors to incorrect detection include greater shoe heel-to-toe drop. Reported recent shoe change, and overall injury rates were lowest among accurate non-rearfoot strikers and predictive of RRI. This information may guide clinicians and coaches on how to manage, rehabilitate and train endurance runners.

## Data Availability

The raw data supporting the conclusions of this article will be made available by the authors, without undue reservation.
